# Superior Outcome of Early ACL Reconstruction versus Initial Non-reconstructive Treatment With Late Crossover to Surgery: A Study From the Swedish National Knee Ligament Registry

**DOI:** 10.1177/03635465211069995

**Published:** 2022-02-02

**Authors:** Emma Bergerson, Kajsa Persson, Eleonor Svantesson, Alexandra Horvath, Jonas Olsson Wållgren, Jon Karlsson, Volker Musahl, Kristian Samuelsson, Eric Hamrin Senorski

**Affiliations:** * Sahlgrenska Sports Medicine Center, Gothenburg, Sweden; † Department of Orthopaedics, Institute of Clinical Sciences, Sahlgrenska Academy, University of Gothenburg, Gothenburg, Sweden; ‡ Department of Orthopaedics, Sahlgrenska University Hospital, Mölndal, Sweden; § Department of Internal Medicine and Clinical Nutrition, Institute of Medicine, Sahlgrenska Academy, University of Gothenburg, Gothenburg, Sweden; ‖ Department of Orthopaedics, NU Hospital Group, Trollhättan, Sweden; ¶ Department of Orthopaedic Surgery, UPMC Freddie Fu Sports Medicine Center, University of Pittsburgh, Pittsburgh, USA; # Department of Health and Rehabilitation, Institute of Neuroscience and Physiology, Sahlgrenska Academy, University of Gothenburg, Gothenburg, Sweden; Investigation performed at University of Gothenburg, Gothenburg, Sweden

**Keywords:** KOOS, PASS, anterior cruciate ligament (ACL), Swedish National Knee Ligament Registry, knee

## Abstract

**Background:**

Although comparable clinical and functional outcomes have been reported after nonsurgical and surgical anterior cruciate ligament (ACL) treatment, few studies have investigated the effects of early versus late ACL reconstruction with initial rehabilitation.

**Purpose:**

To determine patient-reported knee function in patients who initially undergo nonreconstructive treatment after an ACL injury but who later choose to undergo ACL reconstruction as compared with (1) patients undergoing ACL reconstruction close to the index injury and (2) patients treated nonreconstructively at 1 to 10 years of follow-up.

**Study Design:**

Cohort study; Level of evidence, 2.

**Methods:**

Results from the Knee injury and Osteoarthritis Outcome Score (KOOS) were extracted from the Swedish National Knee Ligament Registry for patients treated with nonreconstruction, early ACL reconstruction, and initial nonreconstruction but subsequent ACL reconstruction (crossover group). The KOOS_4_ (a mean of 4 KOOS subscales) was analyzed cross-sectionally at baseline and at the 1-, 2-, 5-, and 10-year follow-ups. Additionally, the Patient Acceptable Symptom State (PASS) was applied to all KOOS subscales from baseline to the 10-year follow-up.

**Results:**

A total of 1,074 crossover, 484 nonreconstruction, and 20,352 early ACL reconstruction cases were included. The crossover group reported lower KOOS_4_ values than the group undergoing early ACL reconstruction at baseline and at all follow-ups (mean difference [95% CI]): baseline, −6.5 (−8.0 to −5.0); 1 year, −9.3 (−10.9 to −7.7); 2 years, −4.8 (−6.3 to −3.2); 5 years, −6.1 (−8.8 to −3.4); and 10 years, −10.9 (−16.3 to −5.2). Additionally, a smaller proportion of the crossover cohort achieved a PASS on KOOS subscales at baseline and through the 1-, 2-, 5-, and 10-year follow-ups as compared with the early ACL reconstruction cohort. No differences were observed between crossover and nonreconstruction cases on either the KOOS_4_ or the PASS at any follow-up.

**Conclusion:**

A greater proportion of patients treated with early ACL reconstruction reported acceptable knee function and superior overall knee function as compared with patients who decided to cross over from nonreconstructive treatment to ACL reconstruction.

An anterior cruciate ligament (ACL) injury can be debilitating and lead to inferior knee function as compared with knee-healthy counterparts, regardless of treatment.^13^ Randomized and nonrandomized studies have reported similar clinical and functional outcomes after surgical and nonsurgical treatment of an ACL injury.^8,9,12,14,20^ Despite this, little is known in terms of how the time from injury to ACL reconstruction affects the patients’ perceived knee function in the short and long term, with the factors that determine which patients might benefit from the different treatment options. Accumulating evidence suggests that patients undergoing delayed ACL reconstruction run the risk of sustaining additional injuries to the knee joint, such as meniscal and cartilage damage, when compared with patients undergoing early ACL reconstruction.^1,5,10,16,18,19^ However, Filbay^4^ proposed that the increased frequency of concomitant injuries in patients undergoing delayed ACL reconstruction could be explained by misdiagnosis or by participation in high-risk activities for months or even years, without undertaking appropriate rehabilitation. Moreover, there is no clear evidence supporting the superiority of ACL reconstruction over rehabilitation in reducing the risk of further knee damage after the index ACL injury.^4^ The primary aim of this study was to compare the patient-reported knee function in patients who were initially treated nonreconstructively after an ACL injury but subsequently opted for ACL reconstruction with (1) those undergoing ACL reconstruction within 1 year of injury and (2) those treated nonsurgically at 1 to 10 years of follow-up using the Swedish National Knee Ligament Registry (SNKLR). The secondary aim was to compare the proportion of patients who reported acceptable knee function among the treatment groups 1 to 10 years after ACL treatment.

## Methods

Prospective registry data were retrieved from the SNKLR, and 3 groups were stratified by the type of treatment: (1) patients who were treated nonreconstructively after an ACL injury but subsequently underwent ACL reconstruction, defined as the crossover group; (2) patients treated nonreconstructively after an ACL injury, defined as the nonreconstruction group; and (3) patients who underwent an ACL reconstruction within 1 year of the ACL injury, defined as the early ACL reconstruction group.

### Study Sample and Eligibility Criteria

Baseline patient demographics, surgery-related factors, and outcomes were extracted from the SNKLR between January 1, 2005, and December 31, 2018, for patients registered with an ACL reconstruction. For patients who crossed over or were treated nonreconstructively, outcome data were extracted for follow-up that occurred between January 1, 2014, and December 31, 2018, owing to database configurations. In the crossover group, only postsurgical data were extracted. Eligible patients were between 15 and 65 years of age with an ACL injury; they also had available data in the SNKLR on patient-reported outcome in terms of Knee injury and Osteoarthritis Outcome Score (KOOS) preoperatively/at baseline or at 1-, 2-, 5-, or 10-year follow-ups. In addition, patients who were registered as having a nonreconstructive treatment after ACL injury in the SNKLR and had available data including Swedish Social Security number, age, sex, and activity at ACL injury were eligible for inclusion and were included in the nonreconstruction or crossover group. Moreover, all patients registered with a unilateral ACL reconstruction within 1 year of ACL injury were eligible for inclusion in the early ACL reconstruction group. Patients were excluded if they had undergone ACL revision or sustained a contralateral knee injury or if they had a vascular injury or nerve injury, associated fracture, or medial or lateral collateral ligament injury registered in the SNKLR. All the patients who did not respond to any patient-reported follow-up were also excluded.

### Swedish National Knee Ligament Registry

The SNKLR is a nationwide database that uses a web-based protocol with the aim of collecting data from ACL reconstructions performed in Sweden (www.aclregister.nu). The registry was established in January 2005, and it comprises data from >50,000 patients who have undergone ACL reconstruction. The SNKLR is estimated to cover >90% of all ACL reconstructions performed in Sweden.^3^ The results from the SNKLR are used to evaluate and compare clinics in Sweden with the aim of developing and improving treatment outcomes. The protocol of the SNKLR consists of 2 parts: 1 surgeon-reported section and 1 patient-reported section. The surgeon indicates activity at injury, time to reconstruction, and information about the applied surgical technique. Previous ipsilateral knee injuries and contralateral or concomitant injuries to the knee, including meniscal and chondral injuries, are also noted. In this study, we grouped the activity at injury into 4 categories: alpine skiing, pivoting sports (eg, soccer, team handball, floorball, and basketball), nonpivoting sports (eg, running, cycling, equestrian sports, and volleyball), and other (eg, traffic accidents and accidents at work or during outdoor life). The patient-reported part comprises demographic variables and outcomes collected at baseline and at 1, 2, 5, and 10 years after ACL reconstruction. The outcome includes the KOOS^17^ and European Quality of Life–Five Dimensions, which evaluate the patient’s perception of treatment outcome. To date, the SNKLR does not systematically collect data from patients who undergo nonreconstructive treatment, although they are invited to register and complete follow-up similar to patients undergoing ACL reconstruction. Patient demographic data are therefore limited to age, sex, and activity at ACL injury. The regional ethical review board in Stockholm, Sweden, approved this study (2011/337-31/3).

### Outcome

The primary outcome of this study was the KOOS, which was cross-sectionally compared across the treatment groups at baseline and at 1-, 2-, 5-, and 10-year follow-ups. The KOOS is a validated self-administered questionnaire consisting of 42 items, summarized in 5 subscales: knee-related Symptoms (7 items), Pain (9 items), Activities of Daily Living (ADL; 17 items), Sport and Recreation (5 items), and knee-related Quality of Life (QoL; 4 items).^17^ The KOOS considers the patient’s perception of knee-related symptoms and function during the previous week. Each subscale is scored from 0 to 100, where 0 indicates the worst possible state and 100 indicates no knee-related symptoms.^17^ Moreover, this study used an additional score created from the KOOS, called the KOOS_4_, which is an average of 4 KOOS subscales, excluding ADL.^6^ The ADL subscale is excluded to avoid a ceiling effect, as a large proportion of patients who sustain an ACL injury are young and active with no limitations in ADL. Finally, the Patient Acceptable Symptom State (PASS) in the KOOS was applied.^15^ A PASS can be calculated from any patient-reported outcome and answers the question of whether the patient is satisfied with the outcome. Threshold values for the KOOS have been suggested by Muller et al^15^ by asking patients who have undergone ACL reconstruction the following question: “Taking account of all the activity you have during your daily life, your level of pain and also your activity limitations and participation restrictions, do you consider the current state of your knee satisfactory?” The PASS thresholds for the KOOS subscales are as follows: Pain, >88.9; knee-related Symptoms, >57.1; ADL, 100; Sport and Recreation, >75.0; and QoL, >62.5.^15^

### Statistical Analysis

All statistical analyses were performed using SAS/STAT (Version 14.2; SAS Institute Inc). Continuous variables were presented as the mean and standard deviation and the median with minimum and maximum values. For categorical variables, the count and proportion were cited. Comparisons were made between the crossover group and (1) the early ACL reconstruction group and (2) the nonreconstruction group. For pairwise comparisons, the Fisher nonparametric permutation test was used for continuous variables. The confidence interval (CI) for the mean difference between groups was also based on the Fisher nonparametric permutation test. The effect size is the difference in the mean and pooled standard deviation. Pairwise comparisons based on dichotomous variables were analyzed using the Fisher exact test (lowest 1-sided *P* value multiplied by 2). The confidence interval for dichotomous variables is the unconditional exact confidence limits. If no exact limits could be computed, asymptotic Wald confidence limits with continuity correction were calculated instead. All statistical tests were 2-sided, and alpha was set at .05.

## Results

A total of 1,074 patients (50.9% women) were identified in the crossover group, of which 577 had an available KOOS at baseline, 588 at 1 year after the ACL reconstruction, 648 at 2 years, 205 at 5 years, and 48 at 10 years. In the early ACL reconstruction group, 20,352 patients were identified, and the corresponding number in the nonreconstruction group was 484. Table 1 shows the preoperative patient demographics for all 3 patient cohorts as well as the time from injury to surgery for the 2 patient cohorts that underwent ACL reconstruction. The activity at ACL injury was most commonly pivoting sports, regardless of the treatment group. The timing from injury to ACL reconstruction in the crossover group was at a mean 1.1 ± 2.0 years, while patients in the early ACL reconstruction group had a mean 0.5 ± 0.2 years.

**Table 1:** Patient Characteristics by Treatment Group^*a*^

	Crossover (n = 1,074)	Nonreconstruction (n = 484)	Early ACL Reconstruction (n = 20,352)
Age, y			
Mean (SD)	27.7 (10.0)	30.6 (11.1)	25.8 (9.5)
Median (range)	25.2 (15.0-63.4)	28.6 (15.1-60.7)	23 (15.0-71.0)
Sex			
Male	527 (49.1)	252 (52.1)	11,446 (56.2)
Female	547 (50.9)	232 (47.9)	8,906 (43.8)
Activity at injury			
Alpine/skiing	128 (11.9)	112 (23.1)	3,019 (14.8)
Pivoting sport	455 (42.4)	221 (45.7)	13,852 (68.1)
Nonpivoting sport	30 (2.8)	17 (3.5)	1,741 (8.6)
Other	155 (14.4)	91 (18.8)	1,709 (8.4)
Undefined	306 (28.5)	43 (8.9)	31 (0.2)
Time from injury to surgery, y, mean (SD)	1.1 (2.0)	—	0.5 (0.2)

a Data are presented as No. (%) unless noted otherwise. ACL anterior cruciate ligament. Dashes indicate not applicable.

### Overall Knee Function: KOOS_4_

**Figure 1.**
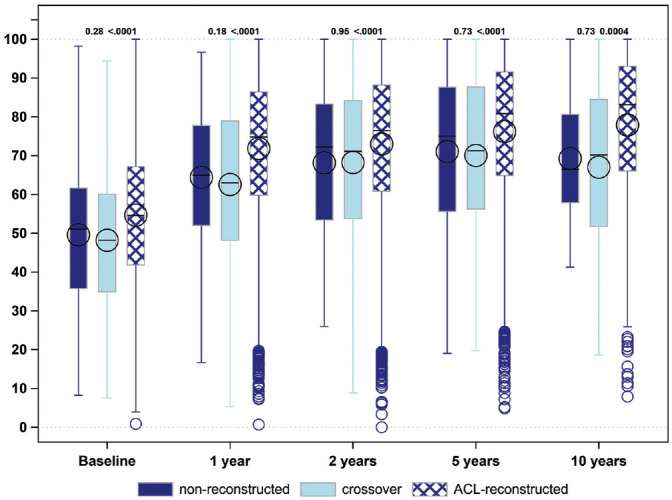
KOOS_4_ score at baseline and 1-, 2-, 5-, and 10-year follow-ups for nonreconstructions, ACL reconstructions, and crossovers. ACL, anterior cruciate ligament; KOOS_4_, Knee injury and Osteoarthritis Outcome Score (average of 4 subscales: knee-related Symptoms, Pain, Sport and Recreation, and knee-related Quality of Life). Line, median; symbol (×, ○), mean; box, interquartile range; error bars, 95% CI; circles, outliers. *P* < .05 is statistically significant.

The crossover group had lower KOOS_4_ scores at baseline as well as follow-up when compared with patients undergoing early ACL reconstruction (mean difference [95% CI]): baseline, −6.5 (−8.0 to −5.0); 1 year, −9.3 (−10.9 to −7.7); 2 years, −4.8 (−6.3 to −3.2); 5 years, −6.1 (−8.8 to −3.4); and 10 years, −10.9 (−16.3 to −5.2). In contrast, there were no differences between the crossover group and the nonreconstruction group in terms of the KOOS_4_ at any follow-up (Table 2, Figure 1).

**Table 2:** KOOS_4_ by Treatment Group^*a*^

				*P* Value^*b*^	
	Crossover (n = 1074)	Nonreconstruction (n = 484)	Early ACL Reconstruction (n = 20,352)	1	2
Baseline					
Mean (SD)	48.2 (17.3)	49.6 (17.7)	54.7 (17.9)	.28	**<.0001**
Median (range)	48.2 (7.5-94.4)	51.1 (8.2-98.2)	54.6 (0.9-100)		
No.	577	253	14,036		
1 y					
Mean (SD)	62.6 (19.7)	64.4 (17.1)	71.8 (18.8)	.18	**<.0001**
Median (range)	63.0 (5.4-100)	65 (16.7-96.7)	74.8 (0.7-100)		
No.	588	284	9,184		
2 y					
Mean (SD)	68.2 (19.5)	68.1 (18.7)	73.0 (19.1)	.95	**<.0001**
Median (range)	71.1 (8.8-100)	72.2 (26-100)	76.5 (0-100)		
No.	648	208	7,680		
5 y					
Mean (SD)	70.1 (20.2)	71.1 (20.0)	76.2 (19.4)	.74	**<.0001**
Median (range)	71.3 (19.7-100)	75 (19-100)	80.8 (4.9-100)		
No.	205	58	4,692		
10 y					
Mean (SD)	67.0 (22.6)	69.2 (16.7)	77.9 (19.2)	.73	**<.001**
Median (range)	70.2 (18.6-100)	66.5 (41.3-100)	83.1 (7.9-100)		
No.	48	16	1,096		

a ACL anterior cruciate ligament, KOOS_4_, Knee injury and Osteoarthritis Outcome Score (average of 4 subscales: knee-related Symptoms, Pain, Sport and Recreation, and knee-related Quality of Life).

b Bold indicates *P* < .05. *P* value 1: crossover vs nonreconstruction. *P* value 2: crossover vs early reconstruction.

### Patient Acceptable Symptom State

**Figure 2.**
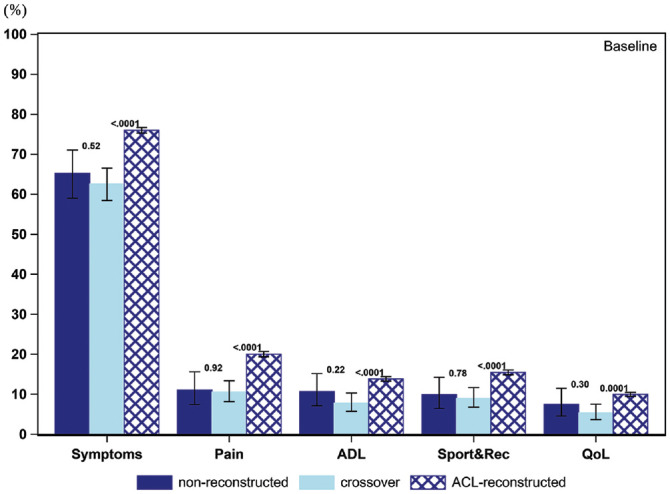
Proportion of patients achieving acceptable knee function at baseline (PASS per KOOS subscale): 253 nonreconstructions, 577 crossovers, and 14,036 ACL reconstructions. ACL, anterior cruciate ligament; ADL, Activities of Daily Living; KOOS, Knee injury and Osteoarthritis Outcome Score; PASS, Patient Acceptable Symptom State; QoL, Quality of Life; Sport&Rec, Sport and Recreation. Error bars indicate 95% CI. *P* < .05 is statistically significant.

**Figure 3.**
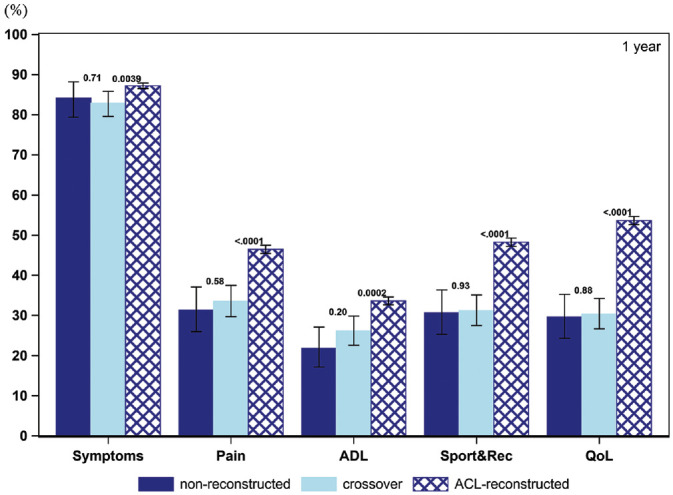
Proportion of patients achieving acceptable knee function at 1-year follow-up (PASS per KOOS subscale): 284 nonreconstructions, 588 crossovers, and 9,184 ACL reconstructions. ACL, anterior cruciate ligament; ADL, Activities of Daily Living; KOOS, Knee injury and Osteoarthritis Outcome Score; PASS, Patient Acceptable Symptom State; QoL, Quality of Life; Sport&Rec, Sport and Recreation. Error bars indicate 95% CI. *P* < .05 is statistically significant.

**Figure 4.**
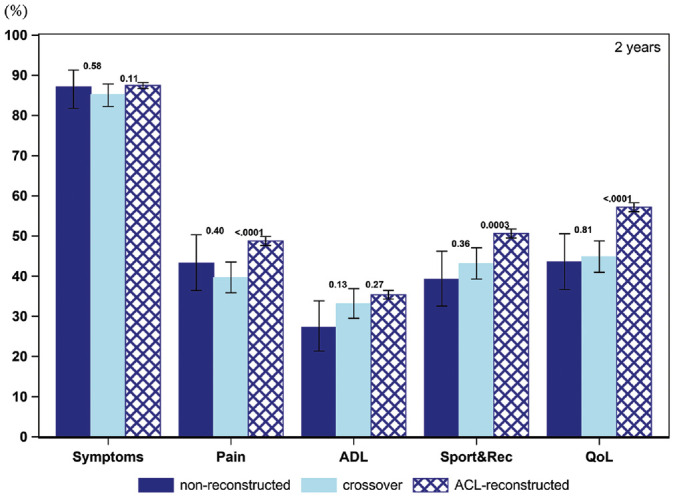
Proportion of patients achieving acceptable knee function at 2-year follow-up (PASS per KOOS subscale): 208 nonreconstructions, 648 crossovers, and 7,680 ACL reconstructions. ACL, anterior cruciate ligament; ADL, Activities of Daily Living; KOOS, Knee injury and Osteoarthritis Outcome Score; PASS, Patient Acceptable Symptom State; QoL, Quality of Life; Sport&Rec, Sport and Recreation. Error bars indicate 95% CI. *P* < .05 is statistically significant.

**Figure 5.**
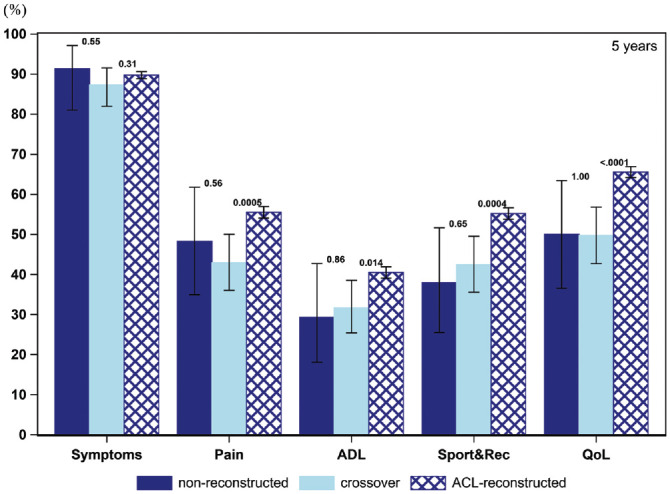
Proportion of patients achieving acceptable knee function at 5-year follow-up (PASS per KOOS subscale): 58 nonreconstructions, 205 crossovers, and 4,692 ACL reconstructions. ACL, anterior cruciate ligament; ADL, Activities of Daily Living; KOOS, Knee injury and Osteoarthritis Outcome Score; PASS, Patient Acceptable Symptom State; QoL, Quality of Life; Sport&Rec, Sport and Recreation. Error bars indicate 95% CI. *P* < .05 is statistically significant.

**Figure 6.**
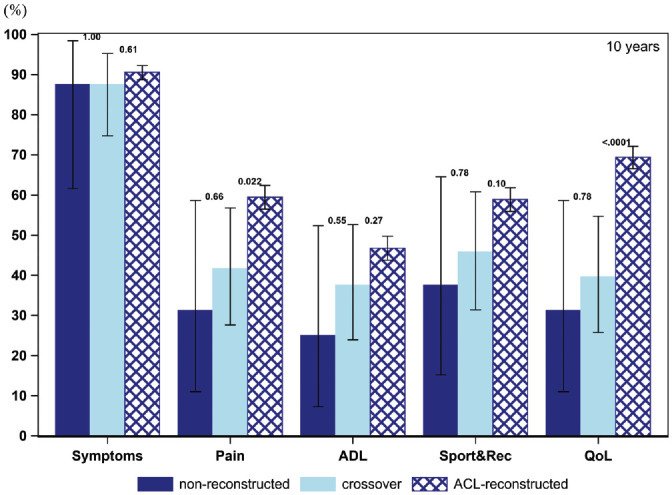
Proportion of patients achieving acceptable knee function at 10-year follow-up (PASS per KOOS subscale): 16 nonreconstructions, 48 crossovers, and 1,096 ACL reconstructions. ACL, anterior cruciate ligament; ADL, Activities of Daily Living; KOOS, Knee injury and Osteoarthritis Outcome Score; PASS, Patient Acceptable Symptom State; QoL, Quality of Life; Sport&Rec, Sport and Recreation. Error bars indicate 95% CI. *P* < .05 is statistically significant.

The PASS for the KOOS items throughout the follow-up for all 3 cohorts is presented in Figures 2 to 6 and Table 3. At baseline, a smaller proportion of patients in the crossover group achieved a PASS for KOOS Symptoms (62.6% vs 76.0%), Pain (10.6% vs 20.0%), ADL (7.8% vs 13.8%), Sport and Recreation (9.0% vs 15.5%), and QoL (5.4% vs 10.0%) as compared with patients treated with early ACL reconstruction. Similarly, in the crossover group, a lower 1-year PASS was noted for all KOOS items as opposed to the early ACL reconstruction group (Symptoms, 82.9% vs 87.2%; Pain, 33.5% vs 46.5%; ADL, 26.1% vs 33.6%; Sport and Recreation, 31.2% vs 48.3%; QoL, 30.3% vs 53.7%). As compared with the early ACL reconstruction group, a smaller proportion of the crossover group met the PASS for the KOOS items of Pain (39.7% vs 48.7%) and Sport and Recreation (43.1% vs 50.6%) but not Symptoms and ADL at the 2-year follow-up. The PASS at 5 years in terms of KOOS Pain (42.9% vs 55.5%), ADL (31.7% vs 40.5%), Sport and Recreation (42.4% vs 55.2%), and QoL (49.8% vs 65.6%) was lower in the crossover group. Yet, no differences were observed in the 5-year PASS for KOOS Symptoms between the crossover and early ACL reconstruction groups. At 10 years, the crossover group had a lower PASS on the KOOS with regards to Pain (41.7% vs 59.5%) and QoL (39.6% vs 69.4%) than patients in the early ACL reconstruction group. However, there were no significant differences between the groups on the KOOS Symptoms, ADL, and Sport and Recreation.

**Table 3:** Patients Reporting Acceptable Knee Function by KOOS Subscale and Treatment Group^*a*^

				*P* Value^*b*^	
KOOS Subscale	Crossover (n = 1,074)	Nonreconstruction (n = 484)	Early ACL Reconstruction (n = 20,352)	1	2
Symptoms					
Baseline	62.6 (361)	65.2 (165)	76.0 (10,668)	.52	**<.0001**
1 y	82.9 (489)	84.2 (239)	87.2 (8,009)	.71	**<.001**
2 y	85.2 (553)	87.1 (182)	87.5 (6,717)	.58	.11
5 y	87.3 (179)	91.4 (53)	89.8 (4,212)	.55	.31
10 y	87.5 (42)	87.5 (14)	90.6 (993)	>.99	.61
Pain					
Baseline	10.6 (61)	11.1 (28)	20.0 (2,810)	.92	**<.0001**
1 y	33.5 (197)	31.3 (89)	46.5 (4,267)	.58	**<.0001**
2 y	39.7 (257)	43.3 (90)	48.7 (3,743)	.40	**<.0001**
5 y	42.9 (88)	48.3 (28)	55.5 (2,604)	.56	**<.001**
10 y	41.7 (20)	31.3 (5)	59.5 (652)	.66	**.02**
ADL					
Baseline	7.8 (45)	10.7 (27)	13.8 (1,940)	.22	**<.0001**
1 y	26.1 (154)	21.8 (62)	33.6 (3,085)	.20	**<.001**
2 y	33.1 (215)	27.3 (57)	35.4 (2,716)	.13	.27
5 y	31.7 (65)	29.3 (17)	40.5 (1,900)	.86	**.01**
10 y	37.5 (18)	25.0 (4)	46.8 (512)	.55	.27
Sport and Recreation					
Baseline	9.0 (52)	9.9 (25)	15.5 (2,168)	.78	**<.0001**
1 y	31.2 (184)	30.6 (87)	48.3 (4,430)	.93	**<.0001**
2 y	43.1 (280)	39.2 (82)	50.6 (3,884)	.36	**<.001**
5 y	42.4 (87)	37.9 (22)	55.2 (2,589)	.65	**<.001**
10 y	45.8 (22)	37.5 (6)	58.9 (645)	.78	.10
QoL					
Baseline	5.4 (31)	7.5 (19)	10.0 (1,398)	.30	**.0001**
1 y	30.3 (179)	29.6 (84)	53.7 (4,927)	.88	**<.0001**
2 y	44.8 (291)	43.5 (91)	57.2 (4,390)	.81	**<.0001**
5 y	49.8 (102)	50.0 (29)	65.6 (3,076)	>.99	**<.0001**
10 y	39.6 (19)	31.3 (5)	69.4 (760)	.78	**<.0001**

a Data are presented as % (No.). ACL, anterior cruciate ligament; ADL, Activities of Daily Living; KOOS, Knee injury and Osteoarthritis Outcome Score; QoL, Quality of Life.

b Bold indicates *P* < .05. *P* value 1: crossover vs nonreconstruction. *P* value 2: crossover vs early reconstruction.

No differences were observed between the crossover and nonreconstructed groups in any PASS for the KOOS at any follow-up.

## Discussion

The principal findings in this study were that patients undergoing early ACL reconstruction had superior overall knee function and a greater proportion reported acceptable knee function, as opposed to those who had crossed over from initial nonreconstructive treatment to late ACL reconstruction. With few exceptions, these differences were consistent from baseline to the 10-year follow-up. When patients from the crossover group were compared with patients assigned to nonreconstruction, no evident differences with respect to knee function were observed at either baseline or the 1-, 2-, 5-, or 10-year follow-ups.

The present study demonstrated superior patient-reported outcome after early ACL reconstruction. It additionally showed that when compared with the crossover group, 12% and >20% more patients in the early ACL reconstruction group had acceptable knee function for the Sport and Recreation and QoL KOOS subscales, respectively, throughout the 10-year follow-up assessments. These findings do not, however, align with previous studies of the topic, such as the Knee Anterior Cruciate Ligament, Nonsurgical versus Surgical Treatment (KANON) trial involving 121 patients,^6,7^ which did not reveal differences in terms of patient-reported knee function (KOOS_4_) between early ACL reconstruction and rehabilitation plus optional delayed ACL reconstruction at 2- and 5-year follow-ups. Although the KANON trial consisted of young patients (age, 18-35 years) who were highly active, the study sample was considerably smaller than that in the present study. As the SNKLR has a coverage of >90% of all ACL reconstructions performed in Sweden,^3^ the present study might represent a more generalizable ACL population. The comparison between reconstructive surgery and nonsurgical treatment of ACL injury was investigated in the prospective Delaware-Oslo ACL Cohort Study^8^ encompassing 143 patients (ACL reconstruction, n = 100; nonsurgical treatment, n = 43). As in the KANON trial, no differences were detected with respect to patient-reported knee function. Additionally, a propensity-scored analysis did not reveal significant differences in the risk of reinjury of the knee between patients assigned to surgical and nonsurgical treatment.^8^

The hypothesis in the present study—that there would be no differences in terms of knee function among the 3 treatment groups—was not confirmed. However, as the purpose of the study was to compare patient-reported knee function at different time points after different treatment options for ACL injury, the study is unable to determine which treatment option is superior after ACL injury. The cross-sectional design precludes conclusions related to differences in treatment effectiveness. Moreover, there was no information about why an individual received a certain treatment, making it impossible to conclude how patients might have evaluated their knee function if another treatment option had been chosen. The patients who underwent early ACL reconstruction reported superior knee function at baseline when compared with patients who crossed over from nonreconstructive treatment to ACL reconstruction. One possible explanation of inferior knee function in patients opting for initial nonreconstructive treatment but subsequent ACL reconstruction versus early ACL reconstruction might be related to subjecting the knee to further damage, especially in the setting of increased laxity.^2,11^ It is important to acknowledge the risk of confounding by indication, as this study is a follow-up of ACL injuries registered in the SNKLR and does not provide information on how or why patients received a specific treatment for their ACL injury. There may also have been differences between treatments and patients that explain the results, such as the level of participation in sport and the amount and quality of rehabilitation.

### Strenghts and Limitations

Important strengths of the study include the assessment of knee function of a large cohort of patients with ACL injury receiving different treatment approaches with a maximum follow-up of 10 years. By focusing on patient-reported outcomes, the patient’s perception of treatment outcome is outlined, and the use of short-, medium-, and long-term follow-up entails the investigation of clinically relevant treatment outcomes over time. Moreover, the cross-sectional analysis maximized the number of patients compared at each follow-up time point. The main limitation of this study is related to the nature of the registry study, which precludes a randomization process to different treatments. This may subject the study to selection bias and possible confounding by indication. There were demographic differences among the treatment groups at follow-up; however, differences had a small effect size and can therefore partly explain the differences in outcomes. Importantly, there was a minimal difference with respect to the time from ACL injury to ACL reconstruction between patients who underwent early ACL reconstruction and patients who crossed over. The reason why some patients had a short time from ACL injury to reconstruction in the crossover group is unknown. The SNKLR does not contain information on patients who initially underwent nonreconstructive treatment but subsequently converted to a surgical treatment approach or how these patients perceived their knee function before ACL reconstruction (ie, when they were treated nonreconstructively). As a result, it was not possible to determine whether patients’ knee function improved after changing treatment strategy from nonsurgical to surgical treatment for their ACL injury. Even though the SNKLR covers approximately >90% of all patients with an ACL reconstruction in Sweden, the coverage of patients treated nonreconstructively is more difficult to assess, as it is the treating physician or physical therapist who gives the patients information about the opportunity to participate in the SNKLR. The inconsistent data collection of nonreonstructively treated patients motivated the decision to start our data extraction from 2014, as the data collection of nonreconstructive data has improved in recent years. However, patients treated nonreconstructively with limited impairment in knee function might not be in contact with the health care system and might thus fail to be enrolled in the registry. There was also no information with respect to concomitant injuries in the crossover and nonreconstruction groups, which might have had an effect on the outcomes. Another possible limitation is the PASS, which was developed for surgically treated patients with a follow-up of 1 to 6 years. In addition, the number of patients in the crossover group was quite small, comprising 48 patients at the 10-year follow-up and thereby limiting the opportunity to draw conclusions from this follow-up.

## Conclusion

This study from the SNKLR shows that a larger proportion of patients treated with early ACL reconstruction reported acceptable knee function and superior overall knee function as compared with patients who were initially treated nonreconstructively and subsequently underwent ACL reconstruction.

## Supplemental Material

sj-pdf-1-ajs-10.1177_03635465211069995 – Supplemental material for Superior Outcome of Early ACL Reconstruction versus Initial Non-reconstructive Treatment With Late Crossover to Surgery: A Study From the Swedish National Knee Ligament Registry Click here for additional data file. Supplemental material, sj-pdf-1-ajs-10.1177_03635465211069995 for Superior Outcome of Early ACL Reconstruction versus Initial Non-reconstructive Treatment With Late Crossover to Surgery: A Study From the Swedish National Knee Ligament Registry by Emma Bergerson, Kajsa Persson, Eleonor Svantesson, Alexandra Horvath, Jonas Olsson Wållgren, Jon Karlsson, Volker Musahl, Kristian Samuelsson and Eric Hamrin Senorski in The American Journal of Sports Medicine
