# Supplementary material for: Superior Outcome of Early ACL Reconstruction versus Initial Non-reconstructive Treatment With Late Crossover to Surgery: A Study From the Swedish National Knee Ligament Registry
Source: Am J Sports Med. 2022 Feb 2;50(4):896–903. doi: 10.1177/03635465211069995 (PMC8980451; doi:10.1177/03635465211069995)
Supplement: sj-pdf-1-ajs-10.1177_03635465211069995 – Supplemental material for Superior Outcome of Early ACL Reconstruction versus Initial Non-reconstructive Treatment With Late Crossover to Surgery: A Study From the Swedish National Knee Ligament Registry [file sj-pdf-1-ajs-10.1177_03635465211069995.pdf]

Early ACL reconstruction results in superior outcome compared with patients who are initially treated non-surgically and cross over to late ACL reconstruction: a study from the Swedish National Knee Ligament Registry

Appendix

Table A1. Demographic comparison between patients in the nonoperatively and crossover group at different follow-ups.

Follow-up	Demographics	Nonoperative group (n=484)	Crossover group (n=1074)	P-value	Difference between groups Mean (95% CI)	Effect Size
**Baseline**		n=254	n=579			
	**Age**	29.6 (10.8)<br>27.2 (15.1; 60)<br>(28.3; 31.0)<br>n=254	27.7 (10.1)<br>25.3 (15; 63.4)<br>(26.9; 28.6)<br>n=579	0.016	1.90 (0.39; 3.43)	0.184
	**Sex**					
	Male	118 (46.5%)	281 (48.5%)		-2.1 (-9.7; 5.6)	0.04
	Female	136 (53.5%)	298 (51.5%)	0.63	2.1 (-5.6; 9.7)	0.04
	**Activity at injury**					
	Alpine/skiing	55 (21.7%)	62 (10.7%)			
	Pivoting sport	115 (45.3%)	235 (40.6%)			
	Non-pivoting sport	10 (3.9%)	13 (2.2%)			
	Other	45 (17.7%)	66 (11.4%)			
	Undefined	29 (11.4%)	203 (35.1%)	<.0001		
	**Time from injury to surgery [years]**		0.385 (0.412)<br>0.307 (0.033; 5.52)<br>(0.351; 0.419)<br>n=579			
**1-year**		n=284	n=590			
	**Age**	30.4 (10.9)<br>28.8 (15.1; 60.7)<br>(29.1; 31.6)<br>n=284	28.0 (10.1)<br>25.5 (15.1; 63.1)<br>(27.2; 28.8)<br>n=590	0.0016	2.36 (0.88; 3.82)	0.228
	**Sex**					
	Male	153 (53.9%)	268 (45.4%)		8.4 (1.1; 15.8)	0.17
	Female	131 (46.1%)	322 (54.6%)	0.023	-8.4 (-15.8; -1.1)	0.17
	**Activity at injury**					
	Alpine/skiing	62 (21.8%)	65 (11.0%)			
	Pivoting sport	140 (49.3%)	230 (39.0%)			
	Non-pivoting sport	10 (3.5%)	16 (2.7%)			
	Other	49 (17.3%)	77 (13.1%)			
	Undefined	23 (8.1%)	202 (34.2%)	<.0001		
	**Time from injury to surgery [years]**		0.689 (0.676)<br>0.557 (0.008; 5.782)<br>(0.635; 0.744)<br>n=590			
**2-year**		n=211	n=650			
	**Age**	31.1 (10.9)<br>29.6 (15.1; 57.4)<br>(29.7; 32.6)<br>n=211	28.3 (10.4)<br>25.4 (15; 63.1)<br>(27.5; 29.1)<br>n=650	0.0003	2.88 (1.24; 4.49)	0.273
	**Sex**					
	Male	107 (50.7%)	295 (45.4%)		5.3 (-2.7; 13.4)	0.11
	Female	104 (49.3%)	355 (54.6%)	0.20	-5.3 (-13.4; 2.7)	0.11

Follow-up	Demographics	Nonoperative group (n=484)	Crossover group (n=1074)	P-value	Difference between groups Mean (95% CI)	Effect Size
	**Activity at injury**					
	Alpine/skiing	45 (21.3%)	86 (13.2%)			
	Pivoting sport	88 (41.7%)	274 (42.2%)			
	Non-pivoting sport	9 (4.3%)	18 (2.8%)			
	Other	47 (22.3%)	91 (14.0%)			
	Undefined	22 (10.4%)	181 (27.8%)	<.0001		
	**Time from injury to surgery [years]**		0.852 (0.806)<br>0.561 (0.008; 5.52)<br>(0.790; 0.914)<br>n=650			
**5-year**		n=58	n=206			
	**Age**	32.6 (11.9)<br>31.2 (15.8; 57.4)<br>(29.4; 35.7)<br>n=58	28.3 (10.8)<br>24.9 (15.3; 63.1)<br>(26.8; 29.8)<br>n=206	0.010	4.27 (1.02; 7.41)	0.386
	**Sex**					
	Male	33 (56.9%)	109 (52.9%)		4.0 (-11.6; 19.5)	0.08
	Female	25 (43.1%)	97 (47.1%)	0.70	-4.0 (-19.5; 11.6)	0.08
	**Activity at injury**					
	Alpine/skiing	22 (37.9%)	26 (12.6%)			
	Pivoting sport	21 (36.2%)	102 (49.5%)			
	Non-pivoting sport	2 (3.4%)	9 (4.4%)			
	Other	10 (17.2%)	31 (15.0%)			
	Undefined	3 (5.2%)	38 (18.4%)	0.0001		
	**Time from injury to surgery [years]**		1.77 (1.99)<br>0.97 (0.01; 14.18)<br>(1.50; 2.05)<br>n=206			
**10-year**		n=17	n=49			
	**Age**	26.6 (9.6)<br>25.1 (15.8; 48.7)<br>(21.7; 31.5)<br>n=17	25.1 (8.1)<br>22.4 (15; 46.6)<br>(22.7; 27.4)<br>n=49	0.54	1.50 (-3.46; 6.15)	0.176
	**Sex**					
	Male	7 (41.2%)	26 (53.1%)		-11.9 (-38.0; 16.4)	0.24
	Female	10 (58.8%)	23 (46.9%)	0.57	11.9 (-16.4; 38.0)	0.24
	**Activity at injury**					
	Alpine/skiing	5 (29.4%)	9 (18.4%)			
	Pivoting sport	10 (58.8%)	20 (40.8%)			
	Non-pivoting sport	0 (0.0%)	3 (6.1%)			
	Other	2 (11.8%)	9 (18.4%)			
	Undefined	0 (0.0%)	8 (16.3%)	0.22		
	**Time from injury to surgery [years]**		6.97 (5.49)<br>7.03 (0.08; 23.97)<br>(5.40; 8.55)<br>n=49			

For categorical variables n (%) is presented.
For continuous variables Mean (SD) / Median (Min; Max) / (95% CI for Mean) / n= is presented.
For comparison between groups Fisher´s Exact test (lowest 1-sided p-value multiplied by 2) was used for dichotomous variables and Chi Square test was used for non-ordered categorical variables and the Fisher´sNon Parametric Permutation Test was used for continuous variable s.
The confidence interval for dichotomous variables is the unconditional exact confidence limits. If no exact limits can be computed the asymptotic Wald confidence limits with continuity correction are calculated
instead The confidence interval for then mean difference between groups is based on Fishers non-parametric permutation test.
Effect size is absolute difference in mean / pooled SD.

Table A2. Comparison of demographics in the crossover and early ACL reconstruction group at different follow-ups.

Follow-up	Demographic	Crossover group (n=1074)	ACL reconstruction group (n=20352)	P-value	Difference between groups Mean (95% CI)	Effect Size
**Baseline**		n=579	n=14044			
	**Age**	27.7 (10.1) 25.3 (15; 63.4) (26.9; 28.6) n=579	25.9 (9.5) 23 (15; 71) (25.7; 26.0) n=14044	<.0001	1.86 (1.06; 2.64)	0.194
	**Sex**					
	Male	281 (48.5%)	7755 (55.2%)		-6.7 (-10.9; -2.4)	0.13
	Female	298 (51.5%)	6289 (44.8%)	0.0018	6.7 (2.4; 10.9)	0.13
	**Activity at injury**					
	Alpine/skiing	62 (10.7%)	2166 (15.4%)			
	Pivoting sport	235 (40.6%)	9563 (68.1%)			
	Non-pivoting sport	13 (2.2%)	1193 (8.5%)			
	Other	66 (11.4%)	1100 (7.8%)			
	Undefined	203 (35.1%)	22 (0.2%)	<.0001		
	**Time from injury to surgery [years]**	0.385 (0.412) 0.307 (0.033; 5.52) (0.351; 0.419) n=579	0.479 (0.238) 0.454 (0; 0.999) (0.475; 0.483) n=14044	<.0001	-0.094 (-0.115; -0.074)	0.382
**1-year**		n=590	n=9322			
	**Age**	28.0 (10.1) 25.5 (15.1; 63.1) (27.2; 28.8) n=590	25.7 (9.7) 22 (15; 71) (25.5; 25.9) n=9322	<.0001	2.30 (1.49; 3.11)	0.236
	**Sex**					
	Male	268 (45.4%)	4712 (50.5%)		-5.1 (-9.4; -0.9)	0.10
	Female	322 (54.6%)	4610 (49.5%)	0.018	5.1 (0.9; 9.4)	0.10
	**Activity at injury**					
	Alpine/skiing	65 (11.0%)	1392 (14.9%)			
	Pivoting sport	230 (39.0%)	6337 (68.0%)			
	Non-pivoting sport	16 (2.7%)	799 (8.6%)			
	Other	77 (13.1%)	776 (8.3%)			
	Undefined	202 (34.2%)	18 (0.2%)	<.0001		
	**Time from injury to surgery [years]**	0.689 (0.676) 0.557 (0.008; 5.782) (0.635; 0.744) n=590	0.490 (0.243) 0.468 (0; 0.999) (0.485; 0.495) n=9322	<.0001	0.199 (0.175; 0.223)	0.694
**2-year**		n=650	n=8186			
	**Age**	28.3 (10.4) 25.4 (15; 63.1) (27.5; 29.1) n=650	26.0 (10.0) 23 (15; 71) (25.8; 26.3) n=8186	<.0001	2.22 (1.42; 3.00)	0.222
	**Sex**					
	Male	295 (45.4%)	4023 (49.1%)		-3.8 (-7.8; 0.3)	0.08
	Female	355 (54.6%)	4163 (50.9%)	0.071	3.8 (-0.3; 7.8)	0.08
	**Activity at injury**					
	Alpine/skiing	86 (13.2%)	1340 (16.4%)			
	Pivoting sport	274 (42.2%)	5482 (67.0%)			
	Non-pivoting sport	18 (2.8%)	693 (8.5%)			
	Other	91 (14.0%)	659 (8.1%)			
	Undefined	181 (27.8%)	12 (0.1%)	<.0001		

Follow-up	Demographic	Crossover group (n=1074)	ACL reconstruction group (n=20352)	P-value	Difference between groups Mean (95% CI)	Effect Size
	**Time from injury to surgery [years]**	0.852 (0.806) 0.561 (0.008; 5.52) (0.790; 0.914) n=650	0.486 (0.242) 0.465 (0; 0.999) (0.481; 0.491) n=8186	<.0001	0.366 (0.340; 0.391)	1.15
**5-year**		n=206	n=5222			
	**Age**	28.3 (10.8) 24.9 (15.3; 63.1) (26.8; 29.8) n=206	26.5 (9.9) 24 (15; 65) (26.2; 26.7) n=5222	0.012	1.81 (0.41; 3.16)	0.183
	**Sex**					
	Male	109 (52.9%)	2536 (48.6%)		4.3 (-2.9; 11.6)	0.09
	Female	97 (47.1%)	2686 (51.4%)	0.25	-4.3 (-11.6; 2.9)	0.09
	**Activity at injury**					
	Alpine/skiing	26 (12.6%)	822 (15.7%)			
	Pivoting sport	102 (49.5%)	3461 (66.3%)			
	Non-pivoting sport	9 (4.4%)	451 (8.6%)			
	Other	31 (15.0%)	474 (9.1%)			
	Undefined	38 (18.4%)	14 (0.3%)	<.0001		
	**Time from injury to surgery [years]**	1.77 (1.99) 0.97 (0.01; 14.18) (1.50; 2.05) n=206	0.500 (0.244) 0.485 (0; 0.999) (0.494; 0.507) n=5222	<.0001	1.27 (1.20; 1.33)	2.80
**10-year**		n=49	n=1257			
	**Age**	25.1 (8.1) 22.4 (15; 46.6) (22.7; 27.4) n=49	26.8 (9.5) 25 (15; 63) (26.2; 27.3) n=1257	0.22	-1.68 (-4.44; 0.96)	0.178
	**Sex**					
	Male	26 (53.1%)	633 (50.4%)		2.7 (-12.6; 18.0)	0.05
	Female	23 (46.9%)	624 (49.6%)	0.82	-2.7 (-18.0; 12.6)	0.05
	**Activity at injury**					
	Alpine/skiing	9 (18.4%)	205 (16.3%)			
	Pivoting sport	20 (40.8%)	822 (65.4%)			
	Non-pivoting sport	3 (6.1%)	124 (9.9%)			
	Other	9 (18.4%)	96 (7.6%)			
	Undefined	8 (16.3%)	10 (0.8%)	<.0001		
	**Time from injury to surgery [years]**	6.97 (5.49) 7.03 (0.08; 23.97) (5.40; 8.55) n=49	0.515 (0.251) 0.501 (0; 0.999) (0.501; 0.529) n=1257	<.0001	6.46 (6.10; 6.76)	5.97

ACL, anterior cruciate ligament.

For categorical variables n (%) is presented.
For continuous variables Mean (SD) / Median (Min; Max) / (95% CI for Mean) / n= is presented.
For comparison between groups Fisher´s Exact test (lowest 1-sided p-value multiplied by 2) was used for dichotomous variables and Chi Square test was used for non-ordered categorical variables and the Fisher´s Non Parametric Permutation Test was used for continuous variables.
The confidence interval for dichotomous variables is the unconditional exact confidence limits. If no exact limits can be computed the asymptotic Wald confidence limits with continuity correction are calculated instead The confidence interval for then mean difference between groups is based on Fishers non-parametric permutation test.

Effect size is absolute difference in mean / pooled SD.
